# Traffic Air Pollution and Other Risk Factors for Respiratory Illness in Schoolchildren in the Niger-Delta Region of Nigeria

**DOI:** 10.1289/ehp.1003099

**Published:** 2011-06-30

**Authors:** B. Adetoun Mustapha, Marta Blangiardo, David J. Briggs, Anna L. Hansell

**Affiliations:** MRC-HPA Centre for Environment and Health, Imperial College London, United Kingdom

**Keywords:** asthma, developing country, indoor air pollution, Niger Delta, outdoor air pollution, respiratory, schoolchildren

## Abstract

Background: Association of childhood respiratory illness with traffic air pollution has been investigated largely in developed but not in developing countries, where pollution levels are often very high.

Objectives: In this study we investigated associations between respiratory health and outdoor and indoor air pollution in schoolchildren 7–14 years of age in low socioeconomic status areas in the Niger Delta.

Methods: A cross-sectional survey was carried out among 1,397 schoolchildren. Exposure to home outdoor and indoor air pollution was assessed by self-report questionnaire. School air pollution exposures were assessed using traffic counts, distance of schools to major streets, and particulate matter and carbon monoxide measurements, combined using principal components analysis. Hierarchical logistic regression was used to examine associations with reported respiratory health, adjusting for potential confounders.

Results: Traffic disturbance at home (i.e., traffic noise and/or fumes evident inside the home vs. none) was associated with wheeze [odds ratio (OR) = 2.16; 95% confidence interval (CI), 1.28–3.64], night cough (OR = 1.37; 95% CI, 1.03–1.82), phlegm (OR = 1.49; 95% CI, 1.09–2.04), and nose symptoms (OR = 1.40; 95% CI, 1.03–1.90), whereas school exposure to a component variable indicating exposure to fine particles was associated with increased phlegm (OR = 1.38; 95% CI, 1.09–1.75). Nonsignificant positive associations were found between cooking with wood/coal (OR = 2.99; 95% CI, 0.88–10.18) or kerosene (OR = 2.83; 95% CI, 0.85–9.44) and phlegm compared with cooking with gas.

Conclusion: Traffic pollution is associated with respiratory symptoms in schoolchildren in a deprived area of western Africa. Associations may have been underestimated because of nondifferential misclassification resulting from limitations in exposure measurement.

Many epidemiologic studies, mainly from developed countries, have shown that exposures to air pollution from ambient particulate matter (PM) pollution and road traffic are associated with respiratory health in children, including upper and lower respiratory symptoms (Bayer-Oglesby et al. 2005; Brunekreef et al. 2009; Kuehni et al. 2006; Künzli et al. 2000; Pierse et al. 2006), changes in pulmonary function (Neuberger et al. 2004), diagnosed asthma (McConnell et al. 2006), medication use (Rabinovitch et al. 2006), and use of medical care (Jalaludin et al. 2004). The evidence of a causal association is strongest for traffic exposure and exacerbations of asthma in children (Health Effects Institute 2010).

Research on health effects of exposures to particulates and traffic pollution in children is sparse in developing countries and particularly scanty in Africa, where exposures are higher than in developed countries (Ezzati et al. 2004). One of the few studies on traffic air pollution and health in Africa comes from an International Study of Asthma and Allergies in Children (ISAAC) analysis (Brunekreef et al. 2009), which suggested dose–response relationships between self-reported truck traffic on the street of residence and current wheeze, rhinoconjunctivitis, and eczema in children 13–14 and 6–7 years of age in two African centers—one in the city of Ibadan, Nigeria, and one in Morocco.

Outdoor PM levels in cities of developing countries including Nigeria are generally much higher than in developed countries because of dispersed heating with small-scale solid fuel use, uncontrolled industrial emissions, and the large numbers of noncatalyst two-stroke engine vehicles. Many vehicles are old and/or poorly maintained, which adds to traffic-related air pollution (Brunekreef 2005). Indoor sources of air pollution also need to be considered, as they can be significant contributors to exposure, particularly in rural areas (World Health Organization and European Commission Joint Research Centre 2002). This study was therefore undertaken to help address the lack of studies in developing countries by testing the hypothesis that ambient and indoor air pollution are risk factors for respiratory illnesses among schoolchildren in Warri, Nigeria, and its environs.

## Methods

*Description of study area.* Warri is a city in Delta state, located in southern Nigeria, an area of lowland rainforest. Intensive oil exploration and production takes place in and around Warri, and it is the location of a major constellation of petrochemical complexes; hence, it is popularly called “oil city.” Outdoor air pollution comes from multiple sources such as oil production and refining in the Niger-Delta region, including gas flaring; refuse and bush burning; and road traffic, largely made up of buses and motorcycles (locally referred to as “okada”) used for public transportation as well as trucks conveying petrol from the Warri refinery to other parts of the country. Vehicles are usually older models originally imported secondhand from developed countries, and there are no exhaust emissions controls. Exposures to indoor air pollution are exacerbated by the use of fuels such as biomass and kerosene, traditional cooking facilities, and poor ventilation.

The Nigerian government is faced with huge developmental challenges. As is true of governments in many developing countries, air quality monitoring and regulation are not a top priority, given the struggle to provide basic necessities such as clean water, food, shelter, basic education, and health care to a rapidly increasing population. In addition, environmental monitoring is hampered by the limited and inconsistent supply of electricity; the difficult terrain of the Niger Delta and frequent political upheavals may also have contributed to a lack of both government and non-government-funded research. Uncertainty about the health impact of ambient air pollution in the region has led to environmental controversies characterized by claims and counter-claims about adverse health effects.

*Study population and study design.* In March–June 2004, a cross-sectional survey was conducted of students 7–14 years of age attending mixed-sex, state primary and secondary schools across 10 municipal areas: Warri, Ebrumede, Effurun, Jeddo, Ubeji, Otor-Udu, Owhrode, Ovu, Udu, and Eku. Twenty of 43 schools in areas identified as being of low socioeconomic status were chosen at random using a metric of 16 indicators specifically developed for the study region (Mustapha 2008). However, three of the schools did not take part in the study because of lack of students of the right age (two schools) or the absence of head teacher (one school).

Students self-completed a written questionnaire in English that included questions about respiratory symptoms based on the ISAAC questionnaire (Ellwood et al. 2000). Preliminary testing indicated that most of the questions required clarification. Questions were therefore read out loud by the interviewer (B.A.M.), who provided additional explanation in Warri Pidgin English. For example, the word “phlegm” was changed to “thick mucus,” and “wheeze” was explained as whistling in the chest. The word “problem” in the ISAAC question “Have you ever had a problem with sneezing, or a runny, or blocked nose when you DID NOT have a cold or the flu?” was changed to “pain,” because pain would be interpreted as discomfort or nuisance, whereas “problem” is interpreted locally as major injury from a fight or something involving the police or military. The question on phlegm production is adapted from the International Union Against Tuberculosis and Lung Disease questionnaire (Burney et al. 1989), in which rainy season is the nearest seasonal equivalent to winter in Nigeria; the other seasons are dry season and harmattan. Other items on the questionnaire concerned demographic and socioeconomic characteristics, respiratory symptoms, perceived sources and levels of indoor air pollution, outdoor exposures, and other risk factors for respiratory illnesses (Mustapha 2008).

Our analysis concentrates on the following five respiratory outcomes:

Wheeze in the previous 12 months (question wording: “Have you had wheezing or noise in your chest at any time in the last 12 months?”)
Night cough in the previous 12 months (“Have you been woken by an attack of coughing at any time in the last 12 months”)
Phlegm (“Do you usually bring up any thick mucus from your chest first thing in the morning in the rainy season?”)
Rhinitis ever (“Have you ever had pain in trying to sneeze or running or blocked nose when you did not have a cold?”)
Doctor-diagnosed asthma ever (“Has a doctor ever told you that you have asthma?”).

Ethical approval for the research protocol was granted by Delta State University, Abraka, Nigeria. Permission was also obtained from the Delta State Ministry of Education, the Delta State Ministry of Health, and local education authorities in the area, as well as head teachers of participating schools. Only students who gave oral informed consent participated in the study.

*Exposure assessment.* Because routine air pollution monitoring in Warri is extremely limited and inadequate as a basis for exposure assessment, and because opportunities for purpose-designed monitoring were constrained by lack of a reliable electricity supply, security issues, and funding limitations, a number of proxy exposure measures were used:

Self-reported indicators of exposure from the questionnaire survey: traffic disturbance (noise and/or fumes) at home, primary home cooking fuel (gas, kerosene, or wood/coal), overcrowding (four or more people sleeping in a room), living with ex- or current smokers, pets with fur or feathers in the home, school nonindustrial pollution (traffic fumes/open burning/cooking smoke), school industrial pollution (gas vapor/flaring), home nonindustrial pollution, and home industrial pollution.
Measurements conducted by one of the authors (B.A.M.) that were combined in principal component analysis (PCA) (King and Jackson 1999) to create an index of air pollution exposure: *a*) distance to major roads—linear distance between the nearest main road and nearest school building; *b*) vehicle counts (categorized as cars, minibuses, trucks, and motorcycles) per 30 min during peak hours on the nearest major road to school (carried out once at each school); *c*) monitored carbon monoxide (CO): a 1-hr average taken three times on the adjacent road, using a hand-held Gastec monitor (Gastec Corporation, Kanagawa, Japan). The three readings were averaged (carried out on two occasions at each school); *d*) monitored PM of various aerodynamic diameters (micrometers), noted below: median mass-equivalent measured with a Dustmate portable environmental monitor (Turnkey Instruments Ltd, Northwich, UK) for 12 to 37 min (mean, 20 min) (carried out on two occasions at each school) for the following fractions: coarse [total suspended particulates (TSP)–PM_10_], intermediate (PM_10_–PM_2.5_), fine (PM_2.5_–PM_1.0_), and very fine (< PM_1.0_)]. Variation in duration of PM measurements was attributable to limitations of battery life of monitor and lack of electricity to recharge battery in the field.

Timing of the monitoring differed between schools and was not concurrent with the questionnaire survey. Measurements at each school were made on weekdays between 1000 and 1400 hours when the schools were in session, and, under sunny weather conditions, when temperatures were between 26 and 31°C. CO and PM were measured twice at each school, and the higher values from each pair of CO or PM readings were used to derive PCA exposure indexes.

We used PCA to create exposure indexes based on measured traffic counts, distance to road, and CO and PM concentrations, using SPSS version 12.0 (SPSS Inc., Chicago, IL, USA). A varimax rotation was performed to maximize independence of the components, and components with eigenvalues > 1.0 were selected (Table 1). This resulted in three exposure components, explaining a total of 78% of the variance. Component 1 appeared to reflect vehicle emissions, because it loaded positively on cars/minibuses, motorcycles, and CO, and inversely on distance from road. Component 2 loaded positively on coarse and intermediate PM and positively on distance from road, possibly representing crustal PM from open ground at greater distance from roads and waste burning. Component 3 showed high positive loadings on fine and very fine PM and on truck traffic and therefore appeared to represent mainly secondary particles derived from more distant (i.e., regional) road and industrial sources.

**Table 1:** Characteristics of factored components.

Factor loadings of rotated principal component				
Variables	Component 1 (vehicle emissions)	Component 2 (coarse particles)	Component 3 (fine particles)
Cars/minibuses		0.963		0.051		0.015
Motorcycles		0.940		0.049		0.053
Trucks		0.453		0.403		0.384
Distance		–0.408		0.314		–0.335
Coarse PM		–0.012		0.899		0.278
Intermediate PM		–0.102		0.887		–0.055
Fine PM		–0.001		0.461		0.850
Very fine PM		–0.060		–0.056		0.960
CO		0.834		–0.285		–0.180
Percent of variance explained		32.135		23.960		22.426
Classification of schools in terms of their air pollution characteristics was carried out by principal components analysis run with a varimax rotation. The table presents the percentage of variance explained by each component and the factor loadings of the variables that constitute the component. Cars/minibuses, motorcycles and trucks were vehicle counts per 30 min during peak hours on the nearest major road to school (carried out once at each school). Distance refers to linear distance between the nearest main road and nearest school building. Coarse PM: total suspended particulates (TSP)–PM_10_; intermediate PM: (PM_10_–PM_2.5_); fine PM: (PM_2.5_–PM_1.0_); very fine PM: < PM_1.0_.						

*Statistical analysis.* Prevalence rates in percentages were computed for the health and exposure variables. We used Student’s *t*-test to determine the presence of significant differences (*p* ≤ 0.05) in measured environmental variables between schools where > 90% of children reported traffic fumes compared with schools where < 10% of children reported traffic fumes as hazard around their schools. We used Pearson correlation analysis to explore the relationship between school CO, PM concentrations, traffic counts, and distance to major roads using SPSS version 12.0.

We used multilevel logistic regression in R (R Project for Statistical Computing, Vienna, Austria) to assess the relationship of the five questionnaire health outcomes (wheeze, night cough, phlegm, rhinitis, and doctor-diagnosed asthma) with the self-reported indoor and outdoor air pollution exposures and the three PCA components (vehicle emissions, coarse particles, and fine particles). Self-reported school exposures (exposure to traffic fumes, open burning, cooking smoke, gas vapor, or gas flaring at school) were not included in the model because they were significantly correlated with factored components and measured CO. Potential confounders included in the models were sex, age, presence of pets in the house, overcrowding, traffic disturbance at home, type of home cooking fuel (wood or coal; kerosene), and presence of smokers in the house. The effect of clustering by school (*n* = 17) and geographic area (north, central, west, south east, south) was explored by including school or school plus area as random effects in the model. Final models were selected as those with the lowest Akaike’s Information Criterion.

## Results

A total of 1,518 children 7–14 years of age were recruited into the survey, and 1,397 (92%) completed usable questionnaires. The median age was 12 years, and 675 (48.3%) were male. Prevalence of the main health outcomes, self-reported potential risk factors in the home, school, and local environments, are shown in Table 2. Night cough (23%), rhinitis (19%), and phlegm (16%) had the highest prevalences in the study group, whereas doctor-diagnosed asthma was rare (0.9%). Practically all children (99%) reported some form of school or home pollution, either in the form of traffic fumes, open bush burning, cooking smoke, gas vapor, or gas flaring at home or school. Traffic disturbance at home was reported by 28% of children. Overcrowding was common, with three-quarters of children (77%) reporting that they slept in a room with at least three other people. Kerosene was the main fuel used for cooking (by 66%), and a third of children (34%) lived in households with a pet.

**Table 2:** Prevalence of household, environmental and five main health variables.

Health and exposure variables	Frequency (no.)	Prevalence (%)	95% CI
Wheeze		76		5.4		4.3–6.6
Night cough		325		23.3		21.1–25.5
Phlegm		232		16.6		14.7–18.6
Rhinitis		268		19.2		17.1–21.3
Diagnosed asthma		12		0.9		0.4–1.3
Home traffic disturbance		390		27.9		25.6–30.3
Cooking fuel: gas		53		3.8		2.8–4.8
Cooking fuel: wood/coal		421		30.1		27.7–32.6
Cooking fuel: kerosene		923		66.1		63.6–68.6
Living with smokers		322		23.1		20.8–25.3
Overcrowding		1,068		76.5		74.2–78.7
Pets		472		33.8		31.3–36.3
School nonindustrial pollution*a*		1,196		85.6		83.8–87.5
School industrial pollution*b*		458		32.8		30.3–35.3
Home nonindustrial pollution*a*		1,105		79.1		77.0–81.2
Home industrial pollution*b*		342		24.4		22.2–26.7
School or home any pollution type		1,387		99.3		98.8–99.7
**a**Nonindustrial pollution: self-reported traffic fumes/open burning/cooking smoke. **b**Industrial pollution: self-reported gas vapor/flaring.						

**Figure 1:**
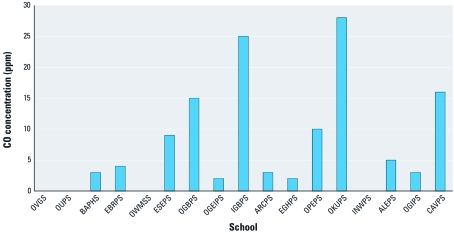
CO concentrations for each school (identified by acronyms). Values shown are means of three readings taken at roads adjacent to school. Four schools (OVGS, OUDS, OWMSS, and INWPS) had zero concentration.

Motorcycles and cars/minibuses dominated the traffic, making up 54% and 44%, respectively, of the total number of vehicles counted. Traffic counts in schools located in urban areas typically had total counts of approximately 2,000 vehicles/hour; schools located in rural areas had 20–60 vehicles per hour. The distance between each school and a major road ranged from 3 to 123 m, with a median of 23 m. Measured CO concentrations at schools ranged from 0 to 28 ppm (Figure 1). The highest CO concentrations were measured at schools located close to major roads, and the lowest concentrations were measured at schools in villages farther from the main road. The mean (± SD) CO concentration for the 12 schools located within mean distance of 27.0 m to a major road was 9.9 ± 9.3 ppm compared with 1.2 ± 1.6 ppm for the five schools located within mean distance of 68.6 m to a major road. Measured CO was strongly correlated with total vehicle counts (*r* = 0.725; *p* = 0.001).

Coarse and intermediate particles dominated the PM fraction in all schools, on average. About 47% of the in-school PM comprised coarse particles, 42% intermediate, 7.4% fine, and 3.6% very fine.

For the multilevel logistic regression, the best-fitting models had a random intercept for school (vs. school plus geographic area) only. Traffic disturbance at home was found to be consistently associated with four of the five health outcomes: wheeze in the previous 12 months [odds ratio (OR) = 2.16; 95% confidence interval (CI), 1.28–3.64], night cough in the previous 12 months (OR = 1.37; 95% CI, 1.03–1.82), phlegm production (OR = 1.49; 95% CI, 1.09–2.04), and rhinitis (OR = 1.40; 95% CI, 1.03–1.90) (Table 3). Almost all of the indoor and outdoor air pollution variables showed positive associations with wheeze, night cough, and phlegm, but none of these reached statistical significance except for the component variable “school fine particles” for phlegm production (OR = 1.38; 95% CI, 1.09–1.75). A household variable, overcrowding, was significantly associated with wheeze (OR = 2.23; 95% CI, 1.11–4.46). There were only 12 cases of doctor-diagnosed asthma, so it is perhaps not surprising that no clear association patterns were seen.

**Table 3:** Multilevel logistic regression analysis of health outcomes and exposure/personal variables [OR (95% CI)].

Exposure/personal variables	Wheeze (previous 12 months)		Night cough (previous 12 months)		Diagnosed asthma (ever)		Phlegm production (rainy season)		Rhinitis (ever)	
Traffic disturbance at home		2.16	(1.28–3.64)^#^		1.37	(1.03–1.82)*		1.63	(0.45–5.94)		1.49	(1.09–2.04)**		1.40	(1.03–1.90)**
Home industrial pollution		1.06	(0.57–1.97)		1.12	(0.83–1.50)		0.49	(0.06–4.31)		0.92	(0.60–1.41)		1.08	(0.79–1.47)
Home nonindustrial pollution		1.15	(0.60–2.21)		1.07	(0.76–1.50)		0.64	(0.10–4.02)		1.33	(0.89–1.99)		0.87	(0.61–1.24)
Primary cooking fuel															
Gas		1.0	(Reference)		1.0	(Reference)		1.0	(Reference)		1.0	(Reference)		1.0	(Reference)
Wood or coal		1.05	(0.27–4.05)		1.53	(0.63–3.70)		0.26	(0.02–4.15)		2.99	(0.88–10.18)		1.29	(0.52–3.19)
Kerosene		0.57	(0.16–2.12)		1.76	(0.75–4.13)		0.13	(0.01–1.78)		2.83	(0.85– 9.44)*		1.26	(0.53 – 3.00)
Component 1		1.34	(0.67–2.70)		1.11	(0.80–1.53)		0.42	(0.11–1.66)		1.15	(0.89–1.49)*		0.91	(0.63–1.32)
Component 2		1.65	(0.83–3.30)		1.02	(0.74–1.40)		0.64	(0.12–3.53)		0.94	(0.73–1.20)		0.84	(0.58–1.21)
Component 3		1.31	(0.67–2.56)		1.05	(0.77–1.44)		0.17	(0.00–6.53)		1.38	(1.09–1.75)**		1.23	(0.86–1.75)
Living with smokers		0.96	(0.53–1.74)		1.11	(0.82–1.49)		0.54	(0.10–2.83)		1.18	(0.84–1.64)		1.12	(0.81–1.55)
Overcrowding		2.23	(1.11–4.46)**		1.02	(0.75–1.39)		1.91	(0.35–10.31)		1.05	(0.74–1.50)		1.34	(0.93–1.91)
Pets		1.02	(0.59–1.75)		1.11	(0.85–1.47)		3.30	(0.87–12.6)*		1.14	(0.83–1.55)		1.23	(0.92–1.66)
Sex		0.90	(0.54–1.51)		1.21	(0.93–1.57)		0.85	(0.24–3.07)		0.89	(0.66–1.19)		0.95	(0.72–1.26)
Age		0.54	(0.31–0.93)**		0.96	(0.70–1.31)		0.85	(0.15–4.96)		1.00	(0.70–1.43)		1.35	(0.94–1.94)
Component 1 denotes school vehicle emissions, component 2 denotes school coarse particles, and component 3 denotes school fine particles. Home traffic disturbance, home industrial pollution, home nonindustrial pollution all compare reported yes with no. Cooking with wood coal and cooking with kerosene are compared with cooking with gas. Component measures: composite measures grouped into that component compared with not in that component. Overcrowding and pets were compared reported yes with no. Sex indicates male compared with female. Age is presented as OR per 1-year increase in age. *0.05 ≤ *p* < 0.1. **0.01 ≤ *p* < 0.05. ^#^*p* < 0.01.															

## Discussion

This study of schoolchildren in low socioeconomic areas of southern Nigeria exposed to multiple sources of outdoor and indoor air pollution suggested that traffic pollution at home was associated with respiratory symptoms of wheeze, cough, phlegm, and rhinitis. Phlegm was also associated with a component variable thought to reflect exposure at school to fine particles from distant sources, although measured concentrations of fine particles were also correlated with the number of trucks outside each school (*r* = 0.476; *p* = 0.053).

Our findings are broadly consistent with other studies that have reported associations between respiratory illnesses in children and proximity to busy roads, especially those traveled by large numbers of trucks (Brunekreef et al. 2009; Ciccone et al. 1998; Hirsch et al. 1999; Janssen et al. 2003; McConnell et al. 2006; Nicolai et al. 2003; Venn et al. 2005), including two of the very few studies involving African populations (Brunekreef et al. 2009; Venn et al. 2005). As previously noted, self-reported truck traffic was associated with asthma symptoms among ISAAC study participants in Nigeria and Morocco (Brunekreef et al. 2009) and “Almost the whole day truck traffic” versus “Never” was associated with wheeze among children 13–14 years of age in three of five African centers (in Morocco, Cote d’Ivoire, Cameroon, and Gabon) that were not included in multivariate analyses because of incomplete data. A study of adults and children in an urban community in Ethiopia (Venn et al. 2005) found that among 3,592 individuals living within 150 m of a road, the risk of wheeze increased significantly in linear relation to proximity to the road (adjusted OR = 1.17 per 30 m proximity; 95% CI, 1.01–1.36). The only other Nigerian study of air pollution and respiratory health in schoolchildren that we identified (Ana et al. 2009) was a pilot study of 400 children in senior secondary schools who were approximately 15 years of age and older (i.e., older than in the present study), conducted in the urban center of Ibadan in western Nigeria. Only descriptive results were presented, and no air quality measurements were conducted. Further, schools were purposely selected to be located near major roadways, so prevalences of self-reported exposures cannot be readily compared with the present study.

A potentially important source of air pollution in the Warri area is the petrochemical industry. Robins et al. (2005) found prior day exposures to both sulfur dioxide and PM_10_ were associated with lower respiratory symptoms among children at a school bordering a refinery in Durban, South Africa. Another South African study found associations between asthma symptoms and exposure to petrochemical refinery emissions in Cape Town estimated based on wind factors and distance of residence from refinery (White et al. 2009).

Reported indoor air pollution was not significantly associated with the outcomes investigated in this study, but phlegm was positively associated with cooking with wood or coal or with kerosene instead of gas. We did not find a relationship between kerosene use and wheeze or rhinitis, in contrast with a study in urban Ethiopia (Venn et al. 2001). We did not see statistically significant associations between living with smokers and cough, phlegm, or wheeze symptoms (Table 3) as might have been expected (Strachan and Cook 1998). However, this result may relate to the question being a poor indicator of indoor air pollution exposures (e.g., because of ventilation in Nigerian houses or smoking habits) or lack of statistical power, or both, rather than a lack of effect.

The low prevalence of diagnosed asthma in this study (0.9%), despite a relatively high prevalence of self-reported symptoms such as wheeze (5.4%), may indicate undiagnosed asthma due to limited access to health services in this low socioeconomic status area. As noted by Burney et al. (1989), recognition of asthma depends on the quality of health services in a study area and, in addition, on readiness of doctors to attach the label “asthma” to asthmalike symptoms. Main priorities for many community health centers in this area of Nigeria are major tropical and life-threatening infectious diseases such as malaria and tuberculosis, and a diagnosis of asthma is likely to indicate either those with severe disease or those who are richer and better able to access health care.

*Limitations of this study.* Because of logistic difficulties and limited resources, individual-level measurements of indoor and outdoor air pollution exposures and modeled air pollution exposures were not available. The self-reported exposures and limited school-based measurements could have led to exposure misclassification, but we consider that this was likely to have been largely nondifferential and therefore to have led to an underestimate of the impacts on health.

Self-reported air pollution exposure may introduce misclassification bias because of individual reporting differences. In a survey of English households, Hunter et al. (2004) found factors such as a person with respiratory disease in the home, belief that pollution is harmful, and socioeconomic status influenced reporting of air pollution exposures. However, other European studies have shown population mean values of reported annoyance with air pollution correlate reasonably well with measured air pollution levels (Forsberg et al. 1997; Jacquemin et al. 2007; Oglesby et al. 2000).

The validity of our survey results is supported by the consistency in exposures reported among children within each school and the consistency of the reported school exposures with measurements taken at each school. For the five main self-reported measures of school-related pollution (traffic, flaring, gas vapor, open burning, and cooking), the concordance among children within schools averaged between 92.5 and 97.7%. In addition, children reporting traffic problems at school were more likely to attend schools sited closer to roads than children who did not. The 12 schools with > 90% of children reporting traffic fumes at school were an average (± SD) of 27.0 ± 27.2 m from the nearest main road, compared with an average distance of 68.6 ± 36.7 m for the five schools where < 10% of children reported traffic-related problems.

Measurements of PM and CO were made only for very short time periods and therefore may not fully reflect usual or long-term exposures. In addition, equipment used for CO measurements was relatively insensitive, and many measurements were close to or below the detection limit. Traffic counts were conducted only once. However, these measurements were not used alone, but in combination with other variables in PCA to estimate a pollution index. The PCA compensates for uncertainties in individual measures, as the weight of evidence is taken from a number of variables.

## Conclusion

In this study, ambient air pollution from traffic had a modest association with respiratory symptoms in schoolchildren in Africa, even after accounting for personal and household risk factors such as overcrowding and use of biomass cooking fuels. There were significant logistical problems conducting this study in a deprived but polluted area of Nigeria, which may have led to an underestimate of the effect size, and findings need to be confirmed in further African studies of this age group. There were very small numbers of children with diagnosed asthma, and an association between traffic pollution and asthma was not demonstrated. However, asthma diagnosis may be an unreliable outcome in similar African settings, as it is partly dependent on access to health care.

## References

[r1] Ana GREE, Shendell DG, Odeshi TA, Sridhar MKC (2009). Identification and initial characterization of prominent air pollution sources and respiratory health at secondary schools in Ibadan, Nigeria.. J Asthma.

[r2] Bayer-Oglesby L, Grize L, Gassner M, Takken-Sahli K, Sennhauser F, Neu U (2005). Decline of ambient air pollution levels and improved respiratory health in Swiss children.. Environ Health Perspect.

[r3] Brunekreef B. (2005). Out of Africa.. Occup Environ Med.

[r4] Brunekreef B, Stewart AW, Anderson HR, Lai CK, Strachan DP, Pearce N (2009). Self-reported truck traffic on the street of residence and symptoms of asthma and allergic disease: a global relationship in ISAAC Phase 3.. Environ Health Perspect.

[r5] Burney PGJ, Laitinen LA, Perdrizet S (1989). Validity and repeatability of the IUATLD (1984) bronchial symptoms questionnaire: an international comparison.. Eur Respir J.

[r6] Ciccone G, Forastiere F, Agabiti N, Biggeri A, Bisanti L, Chellini E (1998). Road traffic and adverse respiratory effects in children. SIDRIA Collaborative Group.. Occup Environ Med.

[r7] Ellwood P, Asher MI, Beasley R, Clayton TO, Stewart AW on behalf of the ISAAC Steering Committee and the ISAAC Phase Three Study Group (2000). International Study of Asthma and Allergies in Children, Phase 3 Manual. ISAAC International Data Centre, Auckland, New Zealand.. http://isaac.auckland.ac.nz/phases/phasethree/phasethreemanual.pdf.

[r8] Ezzati M, Lopez AD, Rodgers A, Murray CJL, eds (2004). Comparative Quantification of Health Risks: Global and Regional Burden of Disease Attribution to Selected Major Risk Factors. Vol 2. Geneva:World Health Organization.. http://www.who.int/publications/cra/en/.

[r9] Forsberg B, Stjernberg N, Wall S. (1997). People can detect poor air quality well below guideline concentrations: a prevalence study of annoyance reactions and air pollution from traffic.. Occup Environ Med.

[r10] Health Effects Institute. HEI Panel on the Health Effects of Traffic-Related Air Pollution (2010). http://pubs.healtheffects.org/view.php?id=334.

[r11] Hirsch T, Weiland SK, von Mutius E, Safeca AF, Grafe H, Csaplovics H (1999). Inner city air pollution and respiratory health and atopy in children.. Eur Respir J.

[r12] Hunter PR, Bickerstaff K, Davies MA (2004). Potential sources of bias in the use of individual’s recall of the frequency of exposure to air pollution for use in exposure assessment in epidemiological studies: a cross-sectional survey.. Environ Health.

[r13] Jacquemin B, Sunyer J, Forsberg B, Götschi T, Bayer-Oglesby L, Ackermann-Liebrich U (2007). Annoyance due to air pollution in Europe.. Int J Epidemiol.

[r14] Jalaludin BB, O’Toole BI, Leeder SR (2004). Acute effects of urban ambient air pollution on respiratory symptoms, asthma medication use, and doctor visits for asthma in a cohort of Australian children.. Environ Res.

[r15] Janssen NAH, Brunekreef B, van Vliet P, Aarts F, Meliefste K, Harssema H (2003). The relationship between air pollution from heavy traffic and allergic sensitization, bronchial hyperresponsiveness, and respiratory symptoms in Dutch schoolchildren.. Environ Health Perspect.

[r16] King JR, Jackson DA (1999). Variable selection in large environmental data sets using principal components analysis.. Environmetrics.

[r17] Kuehni CE, Strippoli MF, Zwahlen M, Silverman M (2006). Association between reported exposure to road traffic and respiratory symptoms in children: evidence of bias.. Int J Epidemiol.

[r18] Künzli N, Kaiser R, Medina S, Studnicka M, Chanel O, Filliger P (2000). Public-health impact of outdoor and traffic-related air pollution: a European assessment.. Lancet.

[r19] McConnell R, Berhane K, Yao L, Jerrett M, Lurmann F, Gilliland F (2006). Traffic, susceptibility, and childhood asthma.. Environ Health Perspect.

[r20] Mustapha BA (2008). A Study of Air Pollution and Respiratory Health in Schoolchildren in Warri, Delta State, Nigeria. [PhD Thesis].

[r21] Neuberger M, Schimek MG, Horak F, Moshammer H, Kundi M, Frischer T (2004). Acute effects of particulate matter on respiratory diseases, symptoms and functions: epidemiological results of the Austrian Project on Health Effects of Particulate Matter (AUPHEP).. Atmos Environ.

[r22] Nicolai T, Carr D, Weiland SK, Duhme H, von Ehrenstein O, Wagner C (2003). Urban traffic and pollutant exposure related to respiratory outcomes and atopy in a large sample of children.. Eur Respir J.

[r23] Oglesby L, Künzli N, Monn C, Schindler C, Ackermann-Liebrich U, Leuenberger P. (2000). Validity of annoyance scores for estimation of long term air pollution exposure in epidemiologic studies: the Swiss Study on Air Pollution and Lung Diseases in Adults (SAPALDIA).. Am J Epidemiol.

[r24] Pierse N, Rushton L, Harris RS, Kuehni CE, Silverman M, Grigg J (2006). Locally generated particulate pollution and respiratory symptoms in young children.. Thorax.

[r25] Rabinovitch N, Strand M, Gelfand EW (2006). Particulate levels are associated with early asthma worsening in children with persistent disease.. Am J Respir Crit Care Med.

[r26] Robins TG, Batterman S, Mentz GB, Kistnasamy B, Jack C, Irusen E (2005). Respiratory health and air pollution in South Durban: the Settlers school study.. Epidemiology.

[r27] Strachan DP, Cook DG (1998). Health effects of passive smoking. 6. Parental smoking and childhood asthma: longitudinal and case-control studies.. Thorax.

[r28] Venn AJ, Yemaneberhan H, Bekele Z, Lewis SA, Parry E, Britton J (2001). Increased risk of allergy associated with the use of kerosene fuel in the home.. Am J Respir Crit Care Med.

[r29] Venn A, Yemaneberhan H, Lewis S, Parry E, Britton J. (2005). Proximity of the home to roads and the risk of wheeze in an Ethiopian population.. Occup Environ Med.

[r30] White N, teWaterNaude J, van der Walt A, Ravenscroft G, Roberts W, Ehrlich R (2009).

[r31] World Health Organization (WHO) and European Commission Joint Research Centre (EC RJC) (2002). Guidelines for Concentration and Exposure-Response Measurement of Fine and Ultra Fine Particulate Matter for Use in Epidemiological Studies (Schwela D, Morawska L, Kotzias D, eds).. http://whqlibdoc.who.int/hq/2002/a76621.pdf.

